# Fire benefits flower beetles in a Mediterranean ecosystem

**DOI:** 10.1371/journal.pone.0198951

**Published:** 2018-06-27

**Authors:** Juli G. Pausas, Josabel Belliure, Eduardo Mínguez, Sergio Montagud

**Affiliations:** 1 Centro de Investigaciones sobre Desertificación (CIDE-CSIC), Montcada, Valencia, Spain; 2 Departamento de Ciencias de la Vida, Universidad de Alcalá, Alcalá de Henares, Madrid, Spain; 3 Ciudadanos por la Ciencia, L'Alfàs del Pi, Alacant, Spain; 4 Institut Cavanilles de Biodiversitat i Biologia Evolutiva, Universitat de València, Paterna, Valencia, Spain; Ecole Pratique des Hautes Etudes, FRANCE

## Abstract

Despite the abundance of plants that benefit from fire in Mediterranean ecosystems, little is known about the possible presence of fire-favoured insects (other than bark beetles). For two years we sampled invertebrates after two large wildfires in eastern Spain and demonstrate that two flower beetle species, *Protaetia morio* and *P*. *oblonga* (*Cetoniidae*), show a pyrophilous behaviour. These beetles were much more numerous after the fires than in unburnt plots around the fire perimeter; in addition, these species tended to increase in number with the distance from the fire perimeter and with fire recurrence, especially *P*. *morio*. These results were maintained for the two postfire years sampled. The results for the beetles do not support the hypothesis of postfire colonization, but that local populations survived the fire as eggs or larvae protected in the soil (endogenous persistence). We propose that the increase in population size (compared with unburnt zones) could be driven by the reduction of their predator populations, as vertebrates that feed on these beetles were disfavoured by fire. That is, the results suggest that these flower beetle species benefit from fire because fire disrupts antagonistic interactions with their predators (predation release hypothesis). Given the omnipresence of small mammals, soil insects, and fires, the processes described here are likely to be general but unexplored.

## Introduction

Fire is an ancient ecological and evolutionary process in many ecosystems [1], and the traits and strategies of plants to cope with recurrent fires are relatively well known [2, 3]. However, this knowledge is more limited for animals [4], and especially for complex networks of interactions that assembles biodiversity [5]. The effects of fire on these interactions, and the cascading effects they may generate, are poorly known. Understanding the role of fire in animal assemblies is important for biodiversity assessments in fire-prone ecosystems [4].

Most plants from fire-prone ecosystems show an endogenous postfire dynamics, that is, populations persist after fire because buds are protected from the fire or because plants have a fire-resistant seed-bank that enables the recruitment of new individuals [2, 6]. The consequence of the prevalence of endogenous dynamics is that, for many mediterranean plants, landscape-scale attributes (e.g., fire size and fire heterogeneity) has relatively little relevance in the postfire regeneration process [2]. In the case of animals, endogenous dynamics is mainly related to microsites acting as refuges [7–9]. However, due to animal mobility, postfire colonisation from adjacent populations is expected to be more relevant than in plants [4]. Consequently, landscape attributes are likely to be important factors determining postfire regeneration and fauna biodiversity. Differing sensitivity to fire between animals and plants suggests that fire can disrupt plant-animal interactions, and in the case of antagonistic interactions, this disruption may have positive effects for one species (the plant) and negative for the other (seed predators [10]). Similar processes may be occurring among animals, for instance, when fire has a more negative effect on predators than on prey.

Among the landscape attributes of special relevance for postfire fauna colonisation and survival is fire size; which is also relevant given the increasingly large fires in recent decades [11, 12]. In large fires, comparing abundance between burnt and unburnt zones, and for burnt areas, between different distances from the fire perimeters (or from unburnt patches), may provide an insight to the regeneration mechanism [13]. For instance, if colonization is the main driver, then presence of the target species would decrease towards the centre of a large burnt area during the early postfire years (negative relation with distance from fire perimeter); if endogenous persistence is the main process, then differences in abundance with distance from the fire perimeter would not be expected; fire-favoured (pyrophillous) species would show not only *in-situ* persistence, but also a higher abundance in the burnt compared to the unburnt areas. Understanding the role of fire-landscape attributes may provide clues about the regeneration and maintenance of fauna diversity in fire-prone ecosystems, given that little is known about these processes, especially in insects [13, 14].

Here we focus on invertebrates because the large diversity of their life forms can be a fruitful source of animal responses, and their fire responses are likely to differ from their predators (vertebrates). Community scale analyses of invertebrate guilds and taxonomic groups show both positive and negative responses depending on the fauna groups and fire characteristics [15–17]. However, little detailed information is available on fire response for specific species, and on the mechanisms behind the responses. Saproxylic and bark insects are probably the best-known species that benefit from fire, especially in temperate and boreal forests [15, 18–20], given that they take advantage of resources generated by fire, such as weakened or dead wood (usually exploited by the insects in their immature stages). Typically, like most xylophages, beetles arrive as adults flying from unburnt areas near the fire, i.e., by colonisation from a neighbouring population. Saproxylic beetles with infrared detectors [21] are of special relevance for their pyrophilly and are one of the few morphological fire-adaptations reported in the animal kingdom [4]. However, little is known about other potentially pyrophilous insects and the mechanisms that could drive increases in population after fire; and even less is known in Mediterranean conditions.

Given that many small vertebrates who feed from invertebrates are likely to be negatively affected by fire [22–25], we predict that there could be insects that benefit from predation release, and thus their population significantly increases after fire. We aim to detect the existence of some specific fire-favoured invertebrates, quantify their response to fire, and reveal the mechanism of population increase in mediterranean shrublands. To do so, we took the opportunity provided by two large high intensity fires (>20000 ha) that occurred in 2012 in eastern Spain. We trapped insects in burnt and adjacent unburnt zones and searched for invertebrates that were abundant in the burnt area. To reveal the mechanism that drives their postfire dynamics, we evaluated to what extent the distance to the fire perimeter and fire recurrence influence the postfire abundance of these species.

## Methods

### Ethics statement

This study complied with the research laws of Spain and the European Union. The protocol of animal capture were approved by the Ethics Committee of the authority responsible of permissions (“*Consellería de Agricultura*, *Medi Ambient*, *Canvi Climàtic i Desenvolupament Rural*” of the Valencia Autonomous Region) with permit number 630/12, 272/13 and 512/14. One of the authors (JB) holds the certificate of competence for designing, supervising and performing animals experiments (category B and C) issued by the “*Consejería de Medio Ambiente y de Orientación del Territorio*” (Madrid, certificate number 10/096442.9/13).

### Study sites

**Fig 1:**
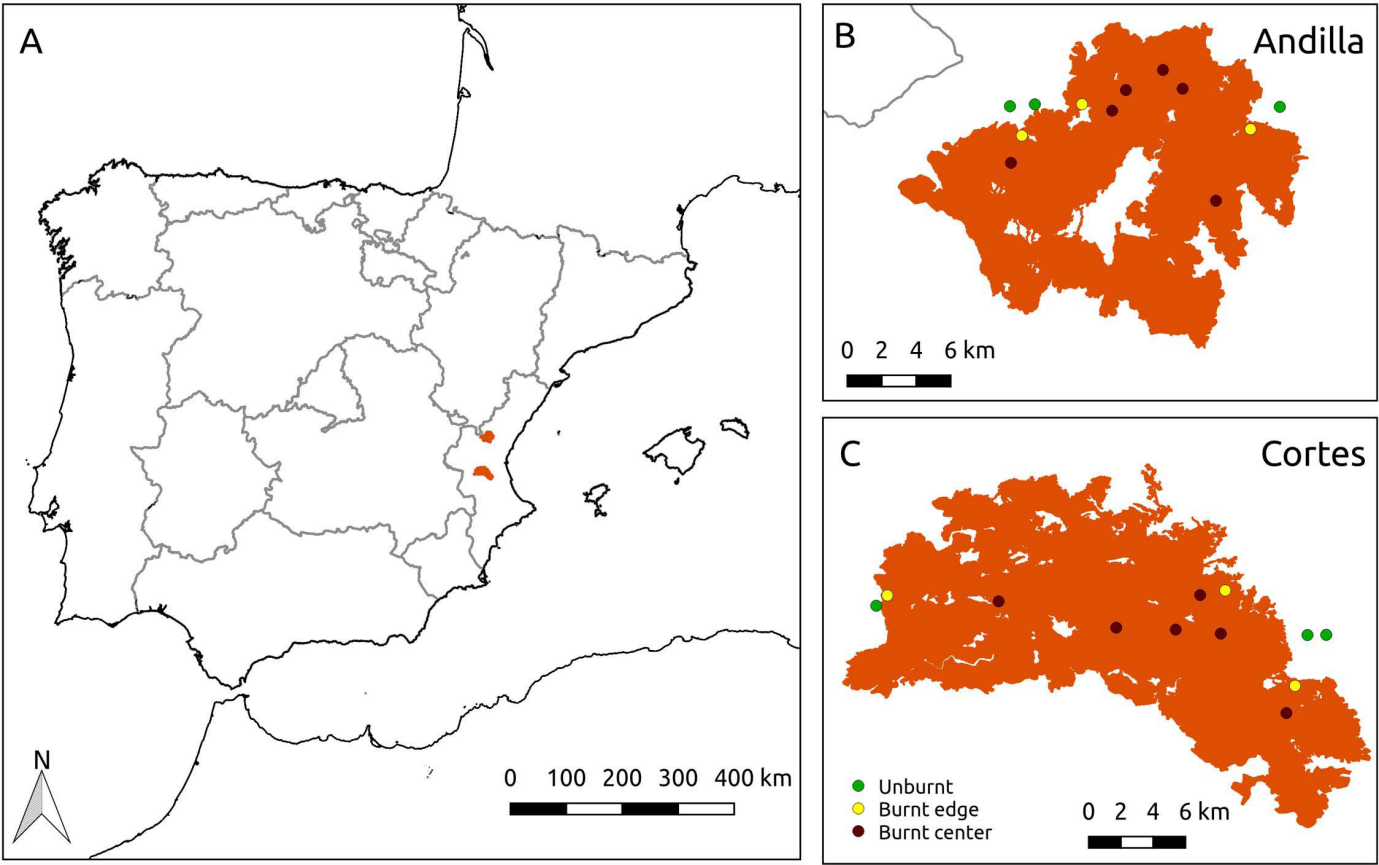
Location of the two study areas at the eastern of the Iberian Peninsula. (A) Location of the two burnt areas (in orange) in the Valencia region, eastern Iberian Peninsula (Andilla is more to the north than Cortes). (B) Andilla burnt area (ca. 21,000 ha). (C) Cortes burnt area (ca. 30,000 ha; Table 1). In A and B, symbols are the location of the sampled plots (green: unburnt; yellow: burnt edge; dark: burnt center). Unburned patches are basically towns, farms and farmland without natural vegetation.

The study was performed in the Valencia region (eastern Spain; Fig 1), an area with a mediterranean climate (S1 Fig) and high fire activity [26, 27]. In June-July 2012, two very large fires (>20000ha) occurred simultaneously and under extreme conditions (hot dry weather with strong winds) in the municipalities of Cortes de Pallás and Andilla (hereafter, Cortes and Andilla fires; Table 1 and Fig 1). Given the weather conditions during the fires, they burnt with a high intensity in most of the fire perimeter, leaving unburnt only towns, farms, roads, and agricultural fields. In most of the area burnt, the fire affected the entire plants (crown-fires), except in some margins of the Andilla fire that were excluded from the sampling. Before the 2012 fires, Cortes was mainly a shrubland dominated by *Q*. *coccifera*, *Cistus sp*. *pl*., *Rosmarinus officinalis*, *Juniperus oxycedrus*, and *Brachypodium retusum*; while in Andilla similar shrublands alternated with pine woodlands (*Pinus halepensis*) and some evergreen oak patches (*Quercus ilex*).

**Table 1:** General characteristics of the study plots in each burnt area.

	Cortes fire	Andilla fire
Fire size (ha)^a^	29752	20945
Elevation (m; mean and range)	441 (190–750)	952 (600–1200)
Distance between plots (Km; range)	1–28	1.4–16
Max distance to fire perimeter (Km)	3.0	2.8
Number of previous fires (1975–2011)	1–4	0
Herb cover (%; Edge vs Center)	10.3±5.9 vs 24.4±13.0	20.2±14.5 vs 29.0±16.0

^a^ Fire size values exclude unburnt patches (towns and farmland); values including unburnt patches are: 31,450 and 23,334 ha, respectively.

The distance between the two fires was 65 km (straight line) and they were located in different mountain ranges and separated by an agricultural valley (where the Lliria meteorological station is located; S1 Fig). The Cortes area is at a lower altitude (Table 1), but the main difference between the two areas is the fire history prior to the 2012 fires. In Cortes, all the area had burnt previously in various fires between 1978 and 1994, and fire recurrence was higher in the centre than at the edges. This is expected because in a mountain surrounded by farmland, even if the fires start at the edges of the mountain, the centre is likely to be more frequently affected by fire than the edges. Consequently, in Cortes, the distance from the fire perimeter is correlated with the fire history. In contrast, the area burnt in the Andilla fire had not been affected by fire during the 1978–2011 period. The size and intensity of these fires and the different fire histories provide an excellent landscape mosaic to study the effect of fire on animals.

### Sampling

For each burnt area (Cortes and Andilla), we selected three plots in the surroundings unburnt area (Unburnt zone), three plots in the burnt area but at less than 700 m from the fire perimeter (Edge zone), and six plots in the centre of the burnt area (Centre zone), that is, plots at more than 1.3 km from the perimeter or from a large unburned patch. So we sampled a total of 24 plots. Because of the large size of the fires, the distance between plots within each fire was considerable (Table 1) and included plots in different watersheds. All plots were selected in zones where the vegetation was shrubland (avoiding areas that were forested before the fire), and where the effect of fire was homogeneous and affected the entire plants (crown-fires). Unburnt plots corresponded to nearby mature shrublands outside the fire perimeter. Because of the intrinsic properties of the two burnt areas, plots were located at a higher altitude in Andilla than in Cortes (Table 1) and they account for the environmental variability of Mediterranean coastal mountains.

In each plot, we set up four pitfall traps (i.e., a total of 96 traps) located in the corners of a 25x25 meter square and a Malaise trap. Pitfall traps were 1L plastic cups (inner diameter of 10 cm at the top and 15.5 cm depth) buried in the soil with the top at ground level, and protected from large vertebrates with a tile a few centimetres above the soil. Traps were filled (ca. 60% of the cup) with propylene glycol (Anorsa, Barcelona). Malaise traps (Entomopraxis S.C.P., Barcelona) consisted of a net of 1.5 x 1 m directing the insects towards a jar with propylene glycol and fixed with ropes and poles.

Traps were set in May 2013 (pitfall and Malaise traps) and 2014 (pitfall traps only), and collected three times each year (2013, 2014) during spring-summer; this sampling provided a total of 576 samples of invertebrates. The number of days between two consecutive samplings in each trap ranged from 19 to 34 with a mean of 25 days (80% of the cases ranged between 21 and 29 days). Note that the spring of 2014 was much drier than the spring of 2013, and drier than most previous years (S1 Fig). We also estimated the vegetation height and cover of each plot in the first postfire year (2013) by visually estimating woody and herbaceous species around each pitfall trap, and then averaging the information at the plot scale.

### Species

Flower beetles (*Cetoniidae* family) were the most abundant insects observed in the samples collected from the traps. Following Paulian (1959) and Báguena (1967), we identified three species of flower beetles: *Oxythyrea funesta* (Poda, 1761), *Protaetia morio* (Fabricius, 1781) and *Protaetia oblonga* (Gory & Percheron, 1833). *P*. *morio* and *P*. *oblonga* were, by far, the most abundant, so we only considered these species in the present study. They were absent or very rare in Malaise traps, and so only pitfall traps were considered in detail. We separated, determined, and counted the number of individuals of these *Protaetia* species in all the samples (Cortes, Andilla). A subset of individuals (*P*. *morio*: n = 239; *P*. *obonga*: n = 254) were also sexed to test whether the pattern observed was driven by females only (e.g., laying eggs).

**Fig 2:**
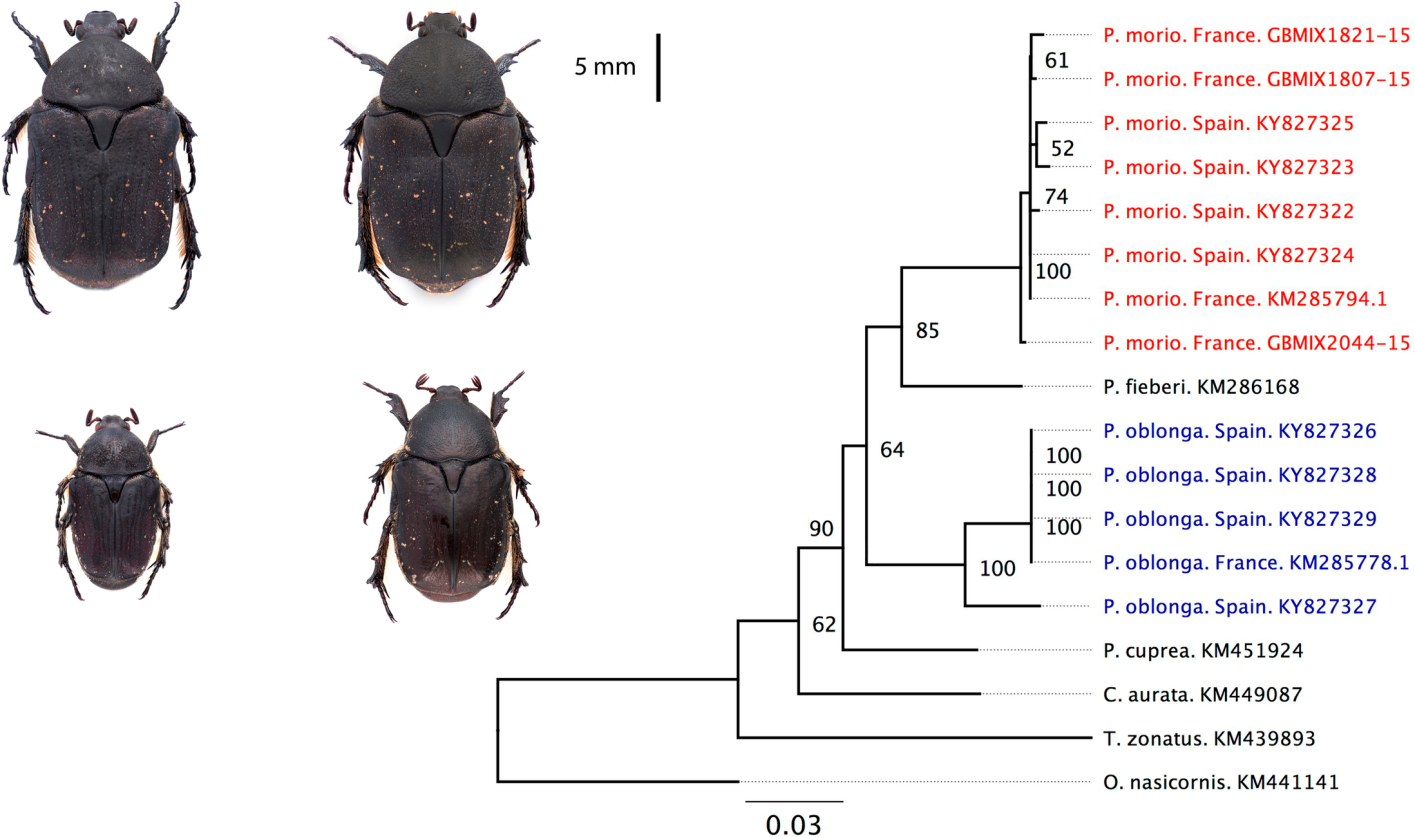
The two species considered (*Protaetia morio P*. *oblonga*) and the Neighbour joining tree of the specimen considered. Left: *Protaetia morio* (upper) and *P*. *oblonga* (lower), showing male (left) and female (right) individuals. Right: Neighbour joining tree of the specimen sampled (those from Spain) plus those obtained from the GenBank and BOLD, including the genus *Protaetia*, *Cetonia*, *Trichius* and *Oryctes*. For details on the methods, see S1 Appendix; for details of the species, see S2 Appendix.

To ensure the specific identity of these two *Protaetia* species and reveal the genetic distances among morphospecies, we used DNA barcoding and assembled a neighbour joining tree (S1 Appendix). The results suggested that the two taxonomic units are two distinct clades and thus supported our morphological identification (Fig 2). All barcoded individuals of *P*. *morio* are genetically very similar, while *P*. *obloga* shows some genetic structure that could reflect some cryptic species, although further taxonomic studies are required (see S1 Appendix for details). For the present study, we used the two morphospecies (distinct clades) as two distinct taxonomic units (*P*. *morio* and *P*. *oblonga*).

These two *Proteatia* species are distributed in the western Mediterranean basin, and are most often found in dry mediterranean habitats. Knowledge of the biology of these species is scare and reviewed in the S2 Appendix. Their life cycle is typically annual. Adults are diurnal flyers and attracted by large flowers. Immature stages (larvae) are typically observed in the soil, in a diverse type of substrate rich in organic matter. In summer, when most fires occur, females have already laid their eggs in the soil. *P*. *oblonga* is typically found in drier and warmer conditions than *P*. *morio* [28, 29] (S2 Appendix). As with other beetles, the main predators of *Protaetia* species are wasps (on larvae [30, 31]), small mammals (larvae and adults [32]), and some birds [33, 34] (S2 Appendix).

### Analyses

The unit of analysis was the plot (averaging the data of the four pitfalls). Our dependent variable (beetle abundance) was standardised to the mean number of individuals accumulated in a pitfall trap during 75 days (three samplings) in a plot (i.e., mean individuals/trap over 75 days).

We defined for each plot, fire recurrence as 'No' (unburnt during the 1978–2012 period), 'Low' (one or two fires during the 1978–2012), and 'High' (three or more fires during the 1978–2012). Because the perimeters of past fires were poorly defined (hand-drawn), a qualitative variable was more appropriate. In Cortes, distance class in the burnt area (Edge vs Centre) and fire recurrence (Low vs High) were correlated (Chi-sq = 5.1, p = 0.025), meaning that the centre of the fire had been burnt more frequently than the edges. While in Andilla, no area was burnt between 1978 and 2012 (see section 'Study sites'; Table 1).

To test the parameters that explained the abundance of each *Protaetia* species we used a linear mixed-model fitted with the maximum log-likelihood approach to abundance (mean individuals/trap over 75 days). Abundance data was log-transformed to improve normality. The selection of variables was based on the lowest AIC (Akaike’s information criterion) for models in which the likelihood ratio test was significant; we used the *nlme* library in R [35]. With this general approach, we performed a set of models as follows: (a) we first independently tested the effect of (i) fire (Burnt vs Unburnt sites), (ii) distance (distance class: Unburnt, Edge, Centre), (iii) fire recurrence (No, Low, High) and (iv) site variables (bare soil, shrub height, herbaceous cover). In each model fire (Cortes, Andilla) and year (2013, 2014) were included as random factors. Then, we fitted combinations of two or more variables to search for the most parsimonious combination of variables (with the lowest AIC). To evaluate whether these parsimonious models were affected by spatial autocorrelation, we compute Moran’s I spatial autocorrelation of the residuals of the model [36] using the NCF software [37]. We then (b) fitted the same models but considering only the plots within the burnt area (plots in the edge and in the center, i.e., excluding plots in the unburnt zone). Thirdly (c), we tested if the two sites (Cortes, Andilla) behave differently in relation to the distance to the perimeter (Distance class) considering Year as random factor. Then (d) we tested the Distance class separated in each of the two sites (Year as random factor). And finally, (e) we tested for differences between years (considering the burnt area, i.e., Cortes/Andilla, as random factor).

## Results

The number of flower beetles (for the two *Protaetia* species together) in each sampling and trap ranged from 0 to 491, with a possible underestimation in the traps with the most individuals as these traps were saturated. Overall, we found 21,808 beetles of the genus *Protaetia*, 63% belonging to *P*. *oblonga* and 36% to *P*. *morio*. The two species occurred in all plots and during both sampled years. On average, the number of individuals of *P*. *oblonga* was higher than *P*. *morio* (mean: 72.3 vs 38.7 individuals/trap/75 days); the variability among plots was much higher in the former species (SD: 120.2, vs 28.2). A small subsample suggested that both males and females were abundant (male to female ratio of about 2:3 in our subsample) and had no effect on the observed pattern.

**Fig 3:**
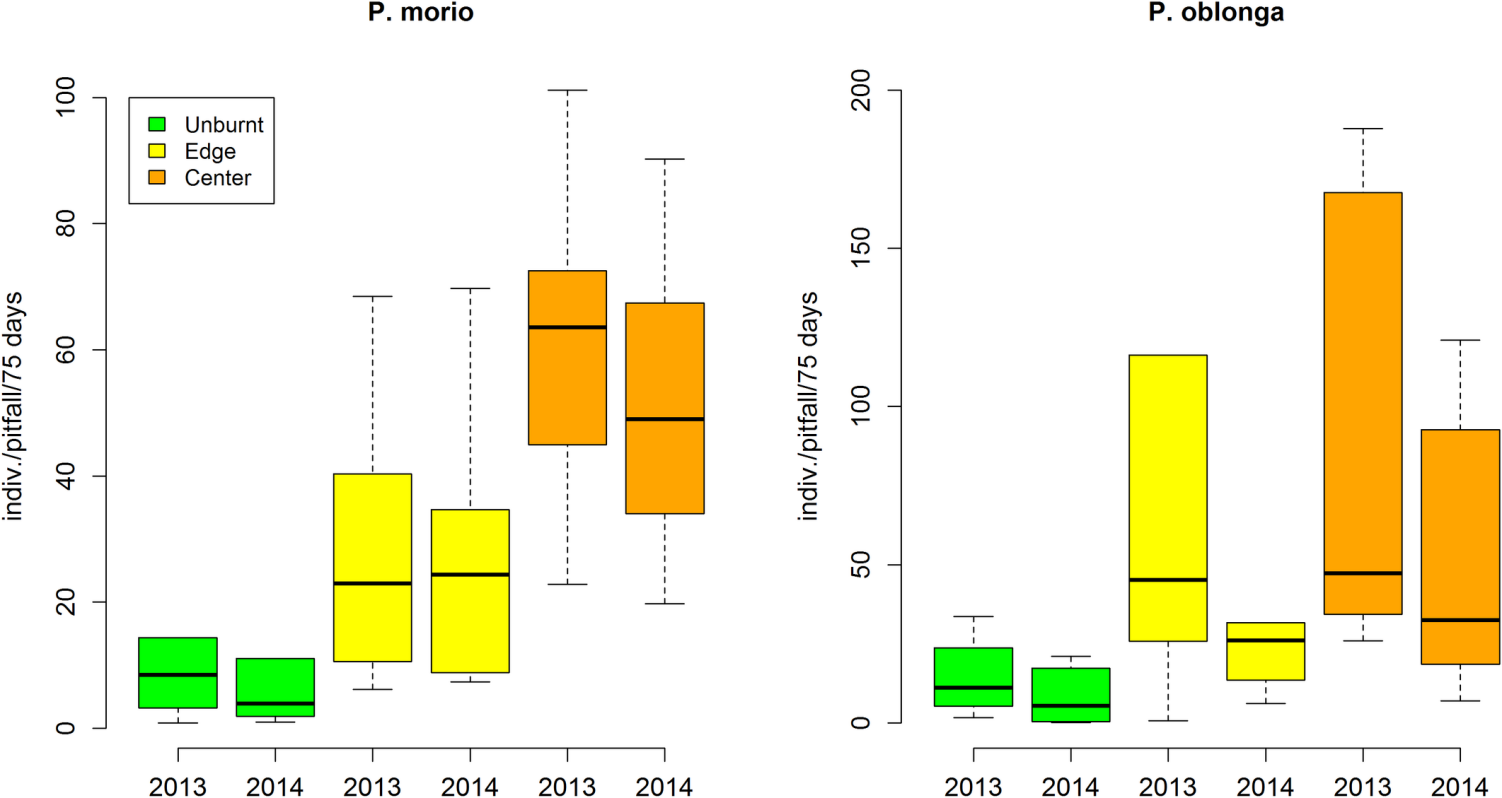
Abundance of *Protaetia morio* (left) and *P*. *oblonga* (right). Values are mean number of individuals accumulated in a pitfall in 75 days for the years 2013 and 2014 in the unburnt area (green), in the burnt area close to the limit with the unburnt area (Edge; yellow) and in the burnt area far from the fire perimeter or from large unburned patches (Centre; orange). Variability is represented by the median (horizontal line), the quartiles (boxes) and the 1.5 times interquartile range (whiskers), and includes between plots and between fires. The number of plots (for each year) is 6, 6 and 12, for Unburnt, Edge and Centre, respectively. See Table 2 for the statistical analysis, and S4 Fig for the data split by the two burnt areas.

For the two species, the abundance was significantly higher in burnt than unburnt plots (Fig 3 and Table 2A). Abundance was also related to the amount of bare soil (positive relation), shrub height (negative relation), distance to the perimeter (higher in the centre, Fig 3), and fire recurrence (higher at high recurrence, S2 Fig). The most parsimonious variable (with the lowest AIC) was Fire Recurrence, closely followed by Distance Class (following the univariate model, Table 1A). Once any of these variables (fire recurrence and distance class) were in the model, Shrub Height also entered in the model for *P*. *morio* (FR+SH in Table 2A); no multivariate model was significant for *P*. *oblonga*. The abundance of *Protaetia* beetles was spatially autocorrelated especially in Cortes (in the two species and for the two years; S1 Table); however, these spatial effects were removed after fitting the most parsimonious model (S1 Table), suggesting that type-I error is unlikely [36].

**Table 2:** Summary of the linear mixed models relating abundance of the two *Protaetia* species with fire and site variables.

	*Protaetia morio*			*Protaetia oblonga*		
Model	AIC^a^	LRT^b^	p	AIC^a^	LRT^b^	p
**a) All plots**						
*Null*	83.86			110.35		
Herbaceous cover [+]	80.90	4.96	0.0259	109.25	3.09	ns
Bare soil [+]	76.50	9.37	0.0022	106.88	5.46	0.019
Shrub height (SH) [–]	57.15	28.72	<0.0001	104.37	7.98	0.0047
Unburned vs Burned	57.97	27.89	<0.0001	94.72	17.62	<0.0001
Distance class	51.35	36.52	<0.0001	95.80	18.54	0.0001
Fire recurrence (FR)	49.10	38.77	<0.0001	83.61	30.73	<0.0001
FR + SH [–]	42.39	8.71	0.032	85.31	0.30	ns
**b) Burned plots only**						
*Null*	26.36			68.25		
Bare soil [+]	26.29	2.06	0.15	68.21	2.03	ns
Shrub height (SH) [–]	25.47	2.89	0.0889	68.16	2.08	ns
Herbaceous cover [+]	23.34	5.02	0.0250	70.24	0.002	ns
Fire recurrence (FR)	21.88	6.49	0.0109	63.20	7.05	0.0079
Distance class	12.78	15.58	0.0001	69.25	0.99	ns
**c) Distance x Site**						
*Null*	81.87			108.29		
Distance class	49.66	36.20	<0.0001	93.37	18.92	0.0001
+ Site	48.25	3.41	ns	94.35	1.02	ns
+ Distance class x Site	39.52	16.14	0.0011	74.24	24.11	<0.0001
**d) Burned only by site**						
*Null* [site = Cortes]	18.10			40.91		
Distance class	6.14	13.96	<0.0002	34.66	8.25	0.0041
*Null* [site = Andilla]	14.22			28.73		
Distance class	12.27	3.95	0.047	26.43	4.30	0.0381
**e) Effect of year**						
*Null*	53.74			88.69		
Year	54.79	0.956	ns	85.04	5.651	0.017

Legend: The five models are: (a) for all plots (burnt and unburnt); (b) for burnt plots only; (c) for all plots, testing the difference between sites in relation with distance; (d) the relation with distance for each site (Cortes, Andilla); and e) for all plots, testing the effect of Year. In (a) we included independent tests (univariate models tested against the corresponding null model) and the most parsimonious multivariate test (last row). In (b) a second variable was never significant, so only univariate models are shown. In (c) the multivariate model is tested sequentially to evaluate the interaction (distance + site + distance x site); site refers to the two burnt areas (Cortes vs Andilla). For (a) and (b) we used Site and Year as random factors; for (c) and (d) Year was the random factor; and for (d) Site and Distance class were random factors. For continuous variables, the sign of the coefficient is given next to the variable in square brackets.

^a^AIC: Akaike information criteria

^b^LRT: likelihood ratio test (ns: p>0.05)

Within the burnt area (i.e., excluding unburnt plots), the results were similar for *P*. *morio*, although many of the variables become non-significant for *P*. *oblonga* (Table 2B). The most parsimonious model for *P*. *morio* was based on Distance (Table 2B). Once either Distance or Fire Recurrence were in the model, no other variables were significant. This relation with distance differed between sites (Table 2C), and was stronger in Cortes than in Andilla (Table 2D and S3 Fig).

*P*. *morio* did not show any changes in abundance between years while *P*. *oblongo* was less abundant in the second year (Year was significant, Table 2E and Fig 3), but the pattern of increasing towards the centre was maintained (no significant interaction).

## Discussion

*Protaetia morio* and *Protaetia oblonga* show a pattern consistent with pyrophilly, that is, their population size largely increases after fire (three and eight times, respectively; Fig 3) and this effect is maintained after two years. The colonization from unburned patches cannot be supported, because under this hypothesis we would not expect more beetles in the burnt than in the unburnt, nor the increasing pattern towards the center. In addition, fires were of high intensity and left very few and small unburned patches (that we avoid in the sampling), and thus it is unlikely they could act as refugia. The large unburned patches were all towns and farmlands (Fig 1), and were considered as the fire perimeter. In any case, fire refugia (including cryptic refugia [38]) could explain the presence of beetles in the burnt area but not more individuals than in the unburnt.

Under the hypothesis of colonization from adjacent populations, we would expect the following: (1) a decreasing abundance towards the centre of the burnt area, at least in the first year (i.e., as increases the distance from the fire perimeter or from the unburned patches); (2) an increased abundance in the burnt area in the second year, especially towards the center; and (3) abundant individuals in the Malaise traps (aerial traps for flying insects). None of these predictions were held by our data. In relation to the first prediction (1), we found higher abundance of the *Protaetia* beetles in the burnt area and a tendency to increase towards the centre (Fig 3), i.e., the pattern opposite to the predicted. In *P*. *morio* this pattern was stronger for both years (Fig 3) and for both burnt areas (Andilla and Cortes; S4 Fig). *P*. *oblonga* was the most abundant species, but also the species that showed most variability among plots and years (Fig 3) which made detecting significant patterns difficult (Table 2). In relation to the second prediction (2), we did not detect any increase in the abundance of *Protaetia* in the second year; in fact the tendency was to decrease, and this tendency was significant for *P*. *oblonga* (Table 2E). The third prediction (3) was not supported either as in 2013, the year in which we set both pitfall and Malaise traps in the same plots, we collected a mean of 127 *Protaetia* beetles per pitfall trap, and plot, and 0.6 per Malaise traps and plot (considering the whole sampling period of 2013 and all plots). Given the intensity of the fires and that none of the above predictions were held by our data, we must conclude that postfire colonisation from nearby unburnt areas was not the dominant process. That is, our results suggest that beetles survived the fire, probably as eggs or larvae protected in the soil. The fact that the pattern is similar for both species and over two years with contrasted climatic conditions (wet spring in 2013 and dry spring in 2014; S1 Fig) further strengthens the result.

**Fig 4:**
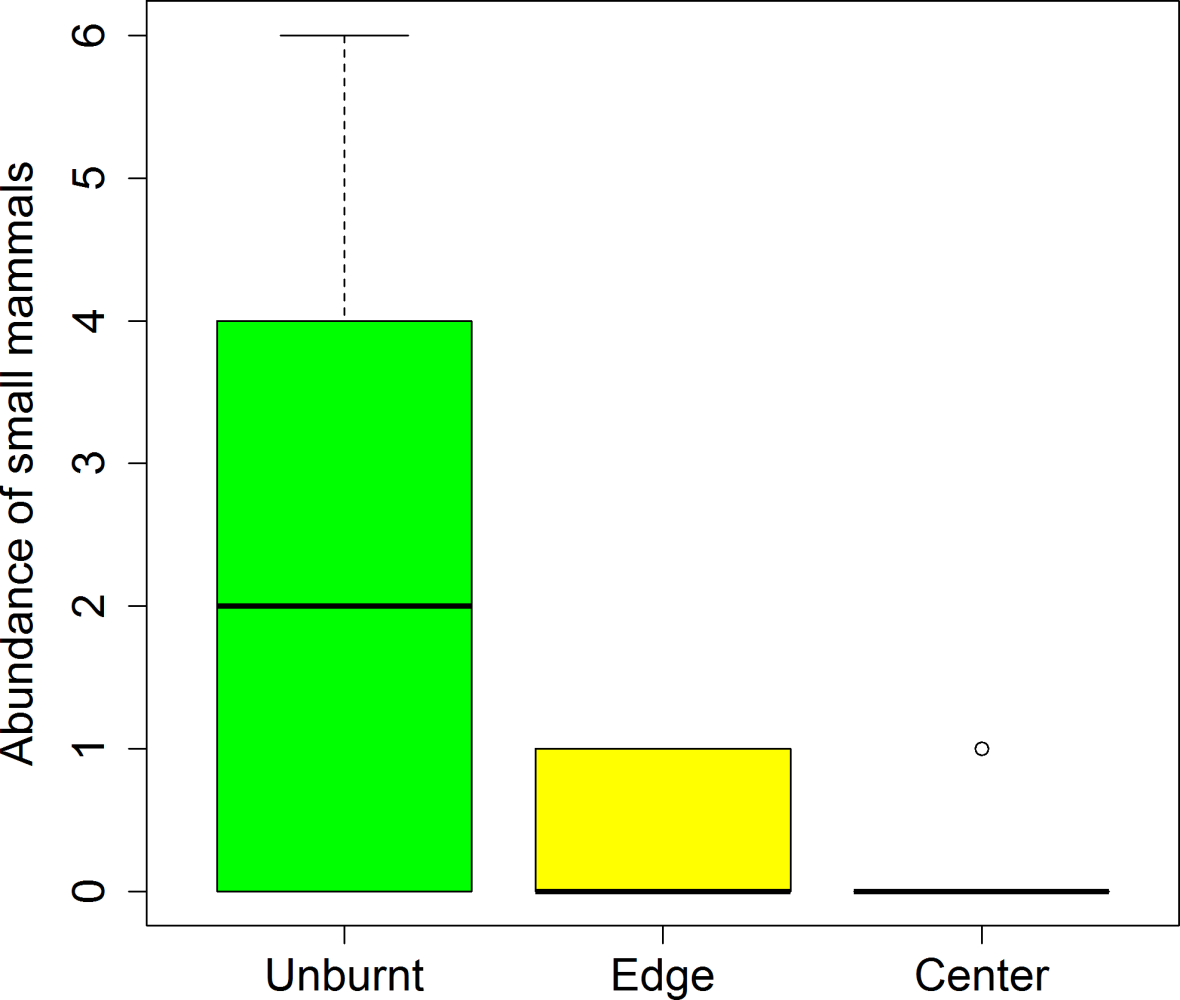
Abundance of small mammals postfire. Mammals collected in pitfall traps during two years (2013, 2014) in Cortes and Andilla, and in three different zones (unburnt, burnt edge and burnt centre). The sampling effort was twice in the center (n = 12 plots) that in the edge (n = 6 plots) and unburnt (n = 6 plots).

However, the question is what drives this seeming pyrophilly? The increasing tendency towards the centre of the fire suggests the existence of a spatially dependent factor interacting with the beetle population. Both distance from the fire perimeter and recurrent fires are candidates as they act in the same direction; they are both positive and significant (Table 2 and Figs 3 and S3). For Cortes, where distance is correlated to fire recurrence, the effect of distance is stronger than in Andilla (where there is no variability in fire recurrence). This suggests that the underlying factor may be relevant to both distance from the fire perimeter (as shown in Andilla) and fire recurrence (as suggested in Cortes). We propose that the most plausible underlying factor is the reduction of predators by fire. *Protaetia* larvae and adults are large and attractive to wildlife, and some of their predators are sensitive to fire. Insect larvae are part of the diet of many small mammals [39–42], including larvae of *Protaetia* species, as they confer fitness benefits to mammals [43, 44]. Looking at the few small mammals that fell in the pitfall traps (*Apodemus sylvaticus*, *Mus spretus*, *Crocidura russula*, *Microtus* sp., *Suncus etruscus*), it was evident that they were much more abundant in the unburnt than in the burnt plots, and almost absent in the centre of the fires (Chi-sq = 42.28, df = 2, p<0.0001, Fig 4). The evidence is especially strong when considering that the sampling effort in the burnt centre zone (n = 12 plots) was twice that in the burnt edge (n = 6) and in the unburnt (n = 6) zones. That is, we found an opposite pattern between the abundance of beetles and the abundance of some of their likely predators (small mammals). Fire can reduce the population of small mammals directly (mortality from the heat and smoke), but also by reducing their habitat quality (e.g., food, shelter [22, 23]). There is plenty of evidence showing that rodents avoid low cover environments due to the high predation risk [45–47]. Thus recently burnt areas are likely to have few active small mammals and thus beetles are released from predation. Mammals can recolonise or temporarily move and feed at the edge of the fire, which would explain the lower abundance in the peripheral areas compared to the centre. In addition, the burnt centre at Cortes has been recurrently burnt and this exacerbates the process, which coincides with the greater significance of the distance in Cortes than in Andilla (Table 2E and S3 Fig). Similar processes could be happening with birds feeding on adult beetles. Consequently, the observed response is consistent with an endogenous persistence, and with a pyrophilly behaviour driven by a predation release.

However, there are other factors that may play a role. For instance, after a fire there may be an increase in the soil organic matter (dead roots) which is the food source for larvae, and this could increase larvae survival and adult numbers. This could contribute to explain the differences between the burnt and unburnt sites, although not the increase towards the centre. In addition, it assumes that soil organic matter is a limiting factor in *Proteatia* populations, which has not yet been demonstrated.

Overall, we provide evidence of an insect with pyrophilous behaviour and endogenous postfire dynamics. The abundance of these flower beetles is consistent with the predation release hypothesis in which fire benefits beetles by disrupting an antagonistic interaction with their predators; however further research is needed to unambiguously demonstrate this process. The consequences of this increase in flower beetles for the ecosystem remain to be studied. In any case, the results contribute to understanding the effect of fire in modifying food webs and interaction networks. Given the omnipresence of small mammals, soil insects, and fires worldwide, the cascading effect described here is likely to be general and unexplored.

## Supporting information

S1 Appendix Barcoding. (PDF)

S2 Appendix Review of the biology of Protaetia (Coleoptera: Cetoniidae). (PDF)

S1 Fig Climate diagram for the llíria meteorological station. Meteorological station located at 250 m asl, between Andilla and Cortes (Valencia province). Bars show the variability in precipitation from 2000 to 2012 (left axis). Symbols are monthly precipitation for the two years sampled (2013, black circles; 2014, white triangles). Line shows mean monthly temperature (^o^C, right axis) for 2000 to 2012 (mean daily temperature averaged by month and year). Variability in temperature among years was much lower (not shown) than for precipitation.
(TIFF)

S2 Fig Abundance of *Protaetia oblonga* and *P*. *morio* by sampling date. Data are mean number of individuals observed in each sampling date and for each pitfall trap (after 25 days in the field). For each species and date, variability refers to the different plots (24) and different years (2 years; except for the first date, April, that was only sampled in the second year, 2014; this April sampling was not considered in the analysis presented in the main text). This data provide an example of coexistence of closely-related species by temporal segregation of the flight activity period. *P*. *oblonga* typically occurs in drier regions than *P*. *morio* (S1 Appendix); in our study area they coexist but the *P*. *oblonga* emerged later, closer to the summer, when the weather was warmer and drier (Fig 1 main text). This temporal partitioning allows coexistence and likely contributes to the maintenance of the diversity of beetles postfire.
(TIFF)

S3 Fig Abundance of *Protaetia morio* (left) and *P*. *oblonga* (right) in relation to fire recurrence. Values are mean number of individuals accumulated in a pitfall in 75 days for 2013 and 2014, in the low and high fire recurrence area (yellow and orange, respectively). Variability includes between plots and between fires; the number of plots (for each year) is 6, 12 and 6, for No, Low and High, respectively. See Table 2 for the statistical analysis. High fire recurrence correspond to plots at the center of the fire, and thus more difficult to colonise by small vertebrates (and thus more depauperated).
(TIFF)

S4 Fig Abundance of *Protaetia morio* (top) and *P*. *oblonga* (bottom) at each site (Andilla and Cortes). Values are mean number of individuals accumulated in a pitfall in 75 days for 2013 and 2014, in the unburnt area (green), in the burnt area close to the limit with the unburnt (Edge, yellow) and in the centre of the burnt area (Center, orange), separated by site (Andilla and Cortes); variability includes among plots and between years; the number of plots (for each year) is 6, 6 and 12, for Unburnt, Edge and Center, respectively. See Table 2 for the statistical analysis.
(TIFF)

S1 Table Spatial autocorrelation. Moran’s autocorrelation values (I and *p*-value) for the abundance of the two *Protaetia* species, the two years and the two sites, and the autocorrelation of the residuals after fitting the most parsimonious model (lowest AIC, Table 2A). Significance is tested by 1000 permutations.
(PDF)

S1 Dataset Data on the abundance of *Protaetia morio* and *P*. *oblonga*. sComma-separated values (csv) file that includes all relevant data of the paper.
(CSV)
