# Supplementary material for: Fire benefits flower beetles in a Mediterranean ecosystem
Source: PLoS One. 2018 Jun 27;13(6):e0198951. doi: 10.1371/journal.pone.0198951 (PMC6021045; doi:10.1371/journal.pone.0198951)
Supplement: S1 Appendix — (PDF) [file pone.0198951.s001.pdf]

S1 Appendix: Barcoding

To ensure the specific identity of the two species of genus *Protaetia*, we used molecular identification through DNA Barcoding which is based on the divergence from part of the COI gene sequence (Hebert et al. 2003). Note that a similarity of 100% of this gene sequence does not guarantee to being in the same taxon, but it reinforces the membership of the same operational taxonomic unit (OTU). A total of eight *Protaetia* individuals from Cortes and Montán (close to Andilla fire) were selected, preserve on absolute ethanol and deposited in the collection of the Universitat de Valencia).

DNA extraction and sequence analysis

For each sample, the medium left leg was extirpated and placed on a 1,5 ml Eppendorf tube with absolute ethanol. Samples were sent for molecular process to AllGenetics & Biology, SL (A Coruña, Spain), where they were analysed as follows: DNA isolations were carried out using the RealPure Microspin Kit (Durviz) following the manufacturer's instructions, and resuspended in a final volume of 30 µL. A negative control that contained no sample was included in every isolation round to check for contamination during the experiments. The primers used for polymerase chain reactions (PCRs) were LCO1490 (5' GGT CAA CAA ATC ATA AAG ATA TTG G) and HCO2198 (5' TAA ACT TCA GGG TGA CCA AAA AAT CA) (Folmer et al. 1994). PCRs were carried out in a final volume of 25 µL, containing 6.50 µL of Supreme NZYTaq Green PCR Master Mix (NZYTech), 0.5 µM of each primer, 2.5 µL of the template DNA solution, and PCR-grade water up to 25 µL. The thermal cycling conditions were as follows: an initial denaturation step at 95 ºC for 5 min, followed by 35 cycles of denaturation at 95 ºC for 30 s; annealing at 50 ºC for 30 s; extension at 72 ºC for 45 s; and a final extension step at 72 ºC for 5 min. A negative control that contained no DNA was included in every PCR round to check for cross-contamination during the experiments. PCR products were run on 1 % agarose gels stained with Real Safe (Durviz), and imaged under UV light. All PCR products yielded one single band of the expected size. PCR products were sequenced using both forward and reverse PCR primers. Electropherogram analysis and overlapping was conducted in Geneious 10.0.3 (Biomatters Ltd). During electropherogram analysis, the primer annealing regions and the low quality regions at both ends of each electropherogram were trimmed (error probability limit of 0.03).

For sequences analysis, all available CO1 sequences from *P. oblonga* (Gory & Percheron, 1833) and *P. morio* (Fabricius, 1781) on GenBank and BOLD (Ratnasingham & Hebert, 2007) were

downloaded. A total of one sequence of *P. oblonga* and four of *P. morio* were obtained (Table S1.1). We also included sequences of the close related species *Cetonia aurata* (Linnaeus, 1761) and *Protaetia fieberi* (Kraatz, 1880). In addition, sequences of *Oryctes nasicornis* (Linnaeus, 1758) and *Trichius zonatus* Germar, 1829 were used as outgroups (Table S1.1). Nucleotide alignment and Neighbor-Joining tree were performed using Geneious 7.1 (Biomatters Ltd), based on Tamura-Nei genetic distance model. Node supports were assessed through 100 bootstrap pseudo-replicates. All eight new sequences obtained from our samples were submitted to GenBank (Table S1.1). Genetic distances between species are reported as minimum uncorrected pairwise distances, while intraspecific variation is reported as maximum uncorrected pairwise distances. DNA extracts are stored at the IVE.

Tabla S1.1. Species, accession code. Length of the sequence, and locality, for the *Protaetia* individuals sampled for barcoding (those from Spain, submitted to GenBank) plus those extracted from GenBank and BOLD. The genus included are: *Cetonia* (Cetoniidae, Cetoniinae), *Protaetia* (Cetoniidae, Cetoniinae), *Oryctes* (Dynastidae, Dynastinae), and *Trichius* (Cetoniidae, Trichiinae).

Species	GenBank	BOLD	Lenght	Locality
Cetonia aurata	KM449087.1		658	France: Provence-Alpes-Cote d'Azur,Var, Draguignan, Le Plan-de-la-Tour, Ortslage
Oryctes nasicornis	KM441141.1		658	Germany: Rhineland Palatinate, Noerdliche Oberrheinebene, Bad Duerkheim, Freinsheim, Kastanienhain Im Hahnen
Protaetia cuprea	KM451924.1		658	Germany: Baden, Suedliches-Oberrhein-Tiefl, Breisgau-Hochschwarzwald, Vogtsburg-Altvogtsburg, Vogelsang
Protaetia fieberi	KM286168.1	PSFOR251-13	658	France: Poitou-Charentes, Deux Sevres, Foret domaniale de Chize
Protaetia morio	KM285794.1	PSFOR245-13	658	France: Languedoc-Roussillon, Pyrenees Orientales, Sorede
Protaetia morio		GBMIX1807-15	658	France: Provence-Alpes-Côte d’Azur, Sérignan-du-Comtat
Protaetia morio		GBMIX1821-15	658	France: Provence-Alpes-Côte d’Azur, Sérignan-du-Comtat
Protaetia morio		GBMIX2044-15	610	France: Languedoc-Roussillon, Montpellier
Protaetia morio	KY827322		658	Spain: Montán, Castellón. Close to Andilla fire.
Protaetia morio	KY827323		658	Spain: Dos Aguas, Valencia. Cortes fire.
Protaetia morio	KY827324		658	Spain: Montán, Castellón. Close to Andilla fire
Protaetia morio	KY827325		658	Spain: Dos Aguas, Valencia. Cortes fire.

Protaetia oblonga	KM285778.1	PSFOR252-13	658	France: Languedoc-Roussillon, Pyrenees Orientales
Protaetia oblonga	KY827326		658	Spain: Montán, Castellón. Close to Andilla fire.
Protaetia oblonga	KY827327		658	Spain: Dos Aguas, Valencia. Cortes forest fire.
Protaetia oblonga	KY827328		658	Spain: Montán, Castellón. Close to Andilla fire.
Protaetia oblonga	KY827329		658	Spain: Dos Aguas, Valencia. Cortes fire.
Trichius zonatus	KM439893.1		658	Germany: Rhineland Palatinate, Ahrtal, Ahrweiler, Grafschaft-Bengen

Results

From the COI phylogenetic tree (Fig. 2 main text) we obtained one well supported clade for all species belonged to *Protaetia* genus. Our two morphological species (*P. morio* and *P. oblonga*) are clearly in distinct clades.

For *P. morio*, the two different populations here studied (Cortes and Andilla-Montán areas), even the French specimens included (from Languedoc-Roussillon and Provence-Alpes-Côte d'Azur departments) show a very constant similarity sequences with no important changes or substitutions. It is important to note that the type locality for *P. morio* is "Europa australi, Italia" (Fabricius, 1781) and the two specimens from Provence-Alpes-Côte d'Azur included are close to the Italian populations to suggest that they belong to the type population of the species. This results show little genetic structure in the French and Iberian Mediterranean populations for *Protaetia morio* and there are no evidences of cryptic species, at least, with the specimens analyzed here.

In contrast, the clades that include the *P. oblonga* specimens show some population structure (Fig. 2 main text). Most of the specimens barcoded have exactly the same sequences, including those from the two areas studied and the one from France. Type locality for *P. oblonga* is "France méridionale" (Gory & Percheron, 1833) and the French specimen included (from Languedoc-Roussillon) comes exactly from this area. This scenario indicates that *Protaetia oblonga* sensu stricto includes populations from France (north) and Valencia (south). But one of our specimen shows a genetic divergence of 4.1 %; this corresponds approximately to four to five million years of isolation from the previous taxon based on typical insect mitocondrial substitution rates (Queck et al. 2014). This specimen does not show any morphological differences from the other *P. oblonga*. This data suggestes the possibility of cryptic species inside the Iberian *P. oblonga* morphospecies, and claim a detailed phylogenetic and morphologic analysis to elucidate this situation. For our present study, we considered *Protaetia oblonga* specimens as a whole (morphospecies).

References (S1 appendix)

Fabricius JC (1781) Species insectorum exhibentes eorum differentias specificas, synonyma, auctorum, loca natalia, metamorphosin. Tome I. Hamburgi et Kilonii

Gory HL, Percheron AR (1833) Monographie des cétoines et genres voisins: formant, dans les familles naturelles de Latreille, la division des Scarabées mélitophiles. J.-B. Baillière

Hebert PDN, Cywinska A, Ball SL, deWaard JR (2003) Biological identifications through DNA barcodes. Proceedings of the Royal Society of London. Series B: Biological Sciences 270:313-321. doi: 10.1098/rspb.2002.2218

Quek S-P, Davies SJ, Itino T, Pierce NE, Pellmyr O (2004) Codiversification in an ant-plant mutualism: stem texture and the evolution of host use in *Crematogaster* (Formicidae: Myrmicinae) inhabitants of *Macaranga* (Euphorbiaceae). Evolution 58:554-570. doi: 10.1554/03-361

Ratnasingham S, Hebert PDN (2007) bold: The Barcode of Life Data System (http://www.barcodinglife.org). Mol. Ecol. Notes 7:355-364. doi: 10.1111/j.1471-8286.2007.01678.x
