# Supplementary material for: Fire benefits flower beetles in a Mediterranean ecosystem
Source: PLoS One. 2018 Jun 27;13(6):e0198951. doi: 10.1371/journal.pone.0198951 (PMC6021045; doi:10.1371/journal.pone.0198951)
Supplement: S2 Appendix — (PDF) [file pone.0198951.s002.pdf]

S2 Appendix: Review of the biology of *Protaetia* (Coleoptera: *Cetoniidae*)

The beetle family Cetoniidae includes over 2.500 species, mostly in tropical and subtropical Africa (Ritcher 1966). Only 49 species belonged to 11 genera are cited from Europe (Jong et al 2014). Adults of the European Cetoniine species are diurnal and good flying insects; they are attracted mainly by the nectar of flowers but they can also eat stamens and pollen (e.g., genus *Oxythyrea* and *Tropinota*). They also feed on the juices of ripening fruits like grapes, apples, peaches, and figs. In some cases, they can become a pest (Ritcher 1966). Copulation takes place during the day and females lay their eggs directly in the substrate that will feed their larvae. The food of Cetoniidae larvae is varied, including flesh organic matter in the soil, piles of discomposing plant material, decaying wood and other debris accumulated in the hollows of trees, pack-rat and rabbit dung and ant nests (Wheeler 1908, 1910; Paulian 1959; Ritcher 1966; Micó & Galante 2003). The life cycle takes between 1-3 years from egg to adult, depending on species and localities.

The genus *Protaetia* Burmeister, 1842, is the most diverse Cetoniine genus of the Palaearctic region, including 55 species (Krajcík 1998), and ca. 250 species worldwide. As other beetles, the main predators of *Protaetia* species are waps (on larvae; Svensson et al. 2012, Schwarz et al. 2013), small mammals (larvae and adults; Lanszki 2003), and some birds (on adults; Overskaug et al. 1995, Fuisz et al. 2013, Hámori 2014, Šumrada & Hanžel 2012, Keckésová & Noga 2008, Kitowski & Pawlega 2010, Saida Tergou et al. 2014).

The two *Proteatia* species considered here (*P. morio* and *P. oblonga*) are distributed in the western Mediterranean basin, and occurs especially in dry mediterranean habitats. Both taxa belong to the subgenus *Netocia* Costa, 1852, that comprises a total of 18 European species (Jong et al. 2014). Despite the relative large size of these beetles and its popularity among entomologists and amateurs, little is know about their biology (Micó & Galante 2003), compared with their more widespread relative, like *Protaetia cuprea* (Fabricius, 1775) and *Protaetia aeruginosa* (Linnaeus, 1767). Following we describe what is known of the biology of the two studied species.

Protaetia (Netocia) morio (Fabricius, 1781)

This species is distributed in North Africa (Morocco, Tunisia and Algeria), Iberian Peninsula, France (reaching the outskirts of Paris and Brittany; Darnaud et al. 1978), southern Switzerland, and Italy (including Sicily and Sardinia) (Baraud 1985).

Adults are active flyers and their activity is always diurnal. They shows flower attraction as other Cetoniidae and has been recorded on flowers of many species, like *Sinapis*, *Verbascum* (Gangloff 1991), *Pyracantha* and *Sambuculus* (Tauzin 2008). They are also common in overripe fruits (Caillol 1913), like figs and other fruits (Paulian 1959; Degallier 1976; Montegud, pers. observ.), and have been also observed in tree wounds (Fauconnet 1887) and in mushrooms (Bedel 1911). There are many records showing that this species is well captured by traps; it was collected on aerial traps filled with red wine or beer and pitfalls setting for Carabidae species (Du Chatenet 1986; Tauzin 2008).

The immature stage is poorly known. Caillol (1913) already suggested that the larvae of *P. morio* lives in the soil close to old trees, mainly willows, where they were frequently infected by the wasp parasite *Scolia hirta* (*Scoliidae*, *Hymenoptera*) (Schrank, 1781). Apparently the larvae do not seems to require any special substrate, as they can develop in a range of conditions with abundant organic matter, including animal faeces (Tauzin 2008); they promote organic matter decay. Devecis (1992) observed females laying eggs even in cricket holes. Larvae can be feed in captivity with a substrate composed of ripe fruits (Micó & Galante 2003); they were also observed feeding on ground grapes in an artificial human habitat (Tauzin 2008).

The life cycle is typically annual, from the egg to adult, but like others Cetoniidae, their metabolism can slow down in cold conditions (Tauzin 2008). Also, the composition amb quality of the organic matter in the food of the larvae can accelerate or delay the development. If larvae feed on ripe fruits they can complete the life cycle in 2-3 months (Micó & Galente, 2003). This species overwinters as adult inside the cocoon or as larvae, probably depending on temperature and organic matter quality. Three instar larvae were described by Micó & Galante (2003). Our data suggest that the peak od the adult activity is in May (Fig. S1).

Protaetia (Netocia) oblonga (Gory & Percheron, 1833)

This species occurs in North Africa, Iberian Peninsula, southern France and Italy (south to Riviera); it is significantly more common in Spain than in France (Bedel 1911); in the latter country it is

mainly restricted to the south-est, below 500 m of altitude, and in hot and dry conditions (Tauzin 2009).

Adults are active diurnal flyers. They feed on flowers, including many thistle species (Tauzin, 2009) like *Cirsium* (Caillol 1913; Paulian 1959), *Centaurea* (Caillol 1913), and *Onopordum* (Tauzin 2009). They also feed on ripe fruits like in mulberries (Paulian & Baraud 1982), plums, figs and apricots (Tauzin 2009; Montegud, pers. observ.). Some few records are from inside beehives, attracted by honey (Moretto 1978). In France, the activity of adults is typically during June and July (Paulian 1959), although there are some records up to November; similar is observed in our Spanish populations (Fig. S1).

Little is known about the immature stage and life cycle. Old records indicate that larvae were found on the ground (Marquet 1898). They can be observed in large numbers near trees on organic material accumulations like fruits or leaves (Tauzin 2009). Some records show also larvae in other specific places rich in organic matter like in abandoned ant nests and rabbit latrines (Micó & Galante 2003). Recent studies indicate that larvae prefer to live around plant root system (Cistaceae, Lamiaceae) with a rich organic soil (Micó & Galante 2003), where they feed from the organic nutrients provided by plant tissues decomposition, and from faecal from animals (Tauzin 2009). In dry environments, larvae are often observed below stones or rocks were the humidity is maintained. There are no evidence on the larval development of *P. oblonga* in tree cavities or other common habitats observed other European *Cetoniinae* species from subgenus different to *Netocia*. The larvae was detailed described by Micó & Galante (2003) and breaded in captivity with a mix of milled rabbit dung and decaying plant matter.

P. oblonga life cycle is typically annual (Tauzin 2009), although there are reports of 2 years life cycle (Micó & Galente 2003). In some northern localities or at higher altitude, metabolism of the larvae can slow down and the life cycle extended (Tauzin 2009). Three instars are observed. The overwinter state seems to be always the larvae (Micó & Galante 2003).

References (appendix S2)

Báguena l (1967) *Scarabaeoidea* de la fauna Ibero-Balear y Pirenaica. Instituto Español de Entomología, CSIC, Madrid

Baraud J (1985) Coléoptères Scarabaeoidea: faune du Nord de l’Afrique du Maroc au Sinaï. Editions Lechevalier, Paris

Baraud J (1992) Coléoptères Scarabaeoidea d'Europe. Société Linnéenne de Lyon, Lyon, FR

Bedel l (1911) Faune des Coléoptères du bassin de la Seine: Scarabaeidae IV (1): 150-152. Société Entomologique de France

Caillol H (1913) Catalogue des coléoptères de Provence (II). Société Linéenne de Provence

Darnaud J, Lecumberry M, Blanc R (1978) Coléoptères Cetoniidae, faune de France. Iconographie entomologique, Scarabaeidae I

Degallier N (1976) Observations sur l'écologie de Cétoines de Corse (Col. Scarabaeidae). Entomologiste 32:32-41

Du Chatenet G (1986) Guide des Coléoptères d'Europe. Delachaux & Niestlé, Paris

Fauconnet l (1887) Catalogue raisonné des Coléoptères de Saône-et-Loire. Société des Sciences Naturelles de Saône-et-Loire

Fuisz TI, Vas Z, Túri K, Kőrösi Á (2013) Photographic survey of the prey-choice of European Bee-eaters (*Merops apiaster* Linnaeus, 1758) in Hungary at three colonies. Ornis Hungarica 21:38-46. doi: 10.2478/orhu-2014-0004

Gangloff l (1991) Catalogue et atlas des Coléoptères d'Alsace. Tome 4-Lamellicornia: Scarabaeidae et Lucanidae. Société Alsacienne d'Entomologie

Hámori D (2014) A kuvik [athene noctua (scopoli, 1769)] táplálkozásának vizsgálata a kiskunságban. Magyar Apróvad Közlemények 12:193-202

Jong Y et al. (2014) Fauna Europaea: all European animal species on the web. Biodiversity Data Journal 2:e4034. doi: 10.3897/BDJ.2.e4034

Kečkéšová L, Noga M (2008) The diet of the Common Kestrel in the urban environment of the city of Nitra Slovak Raptor Journal, vol. 2, p 81

Kitowski I, Pawlega K (2010) Food composition of the Little Owl Athene noctua in farmland areas of South East Poland. Belg. J. Zool. 140:203-211

Krajčík M (1998) Cetoniidae of the World. Catalogue- Part I (Coleoptera: Cetoniidae). Typos Studio Most, Czech Republic

Lanszki J (2003) Feeding habits of stone martens in a Hungarian village and its surroundings. Folia Zoologica 52:367-377

Marquet C (1898) Catalogue des Coléoptères du Languedoc, Toulouse

Micó E, Galante E (2003) Larval morphology and biology of four *Netocia* and *Potosia* species (*Coleoptera*: *Scarabaeoidea*: *Cetoniidae*: *Cetoniinae*). Eur. J. Entomol. 100:131-142

Moretto PH (1978) Contribution à la connaissance de la faune entomologique du Var. Deuxième partie (suite). Lamellicornia (Annales de la Société des Sciences Naturelles et d'Archéologie de Toulon et du Var) 30:140-154

Overskaug K, Kristiansen E, Sunde P (1995) Sex-specific diet analysis of the Tawny Owl *Strix aluco* in Norway. Journal of Raptor Research 29:137–140

Paulian R (1959) Faune de France 63: Coléoptères Scarabéides (2nd ed). Editions Lechevalier, Paris

Paulian R, Baraud J (1982) Faune des Coléoptères de France, Lucanoidea et Scarabaeoidea (II). Paris, Éditions Lechevalier

Ritcher PO (1966) White Grubs and their allies. A Study of North America Scarabaeoid Larvae. Oregon State Monographs. Studies in Entomology, 4

Šumrada T, Hanžel J (2012) The Kestrel *Falco tinnunculus* in Slovenia–a review of its distribution, population density, movements, breeding biology, diet and interactions with other species. Acrocephalus 33:5-24

Tauzin P (2008) Chorologie et étho-écologie de *Protaetia* (*Netocia*) *morio* Fabricius 1781 sur le territoire français (*Coleoptera, Cetoniinae, Cetoniini*). Cetoniimania (Bulletin de l'Association Planète Cétoines) 5:3-40

Tauzin P (2009) Chorologie et éco-éthologie de *Protaetia* (*Netocia*) *oblonga* Gory et Percheron 1833 en France (*Coleoptera, Cetoniinae, Cetoniini*). Cetoniimania (Bulletin de l'Association Planète Cétoines) 6:31-48

Tergou S, Boukhemza M, Marniche F, Milla A, Doumandji S (2014) Dietary distinctive features of Tawny Owl *Strix aluco* (Linn 1758) and Barn Owl *Tyto alba* (Scopoli 1759) in gardens of Algerian Sahel El Harrach Jardin D'essai Du Hamma. Pak. J. Zool. 46:1013-1022

Wheeler WM (1908) Studies on Myrmecophiles. I. Cremastochilus. J. N. Y. Entomol. Soc. 16:68-79

Wheeler WM (1910) Ants: their structure, development and behavior. Columbia University Press, NY
