# Supplementary figures and images for: Fire benefits flower beetles in a Mediterranean ecosystem
Source: PLoS One. 2018 Jun 27;13(6):e0198951. doi: 10.1371/journal.pone.0198951 (PMC6021045; doi:10.1371/journal.pone.0198951)

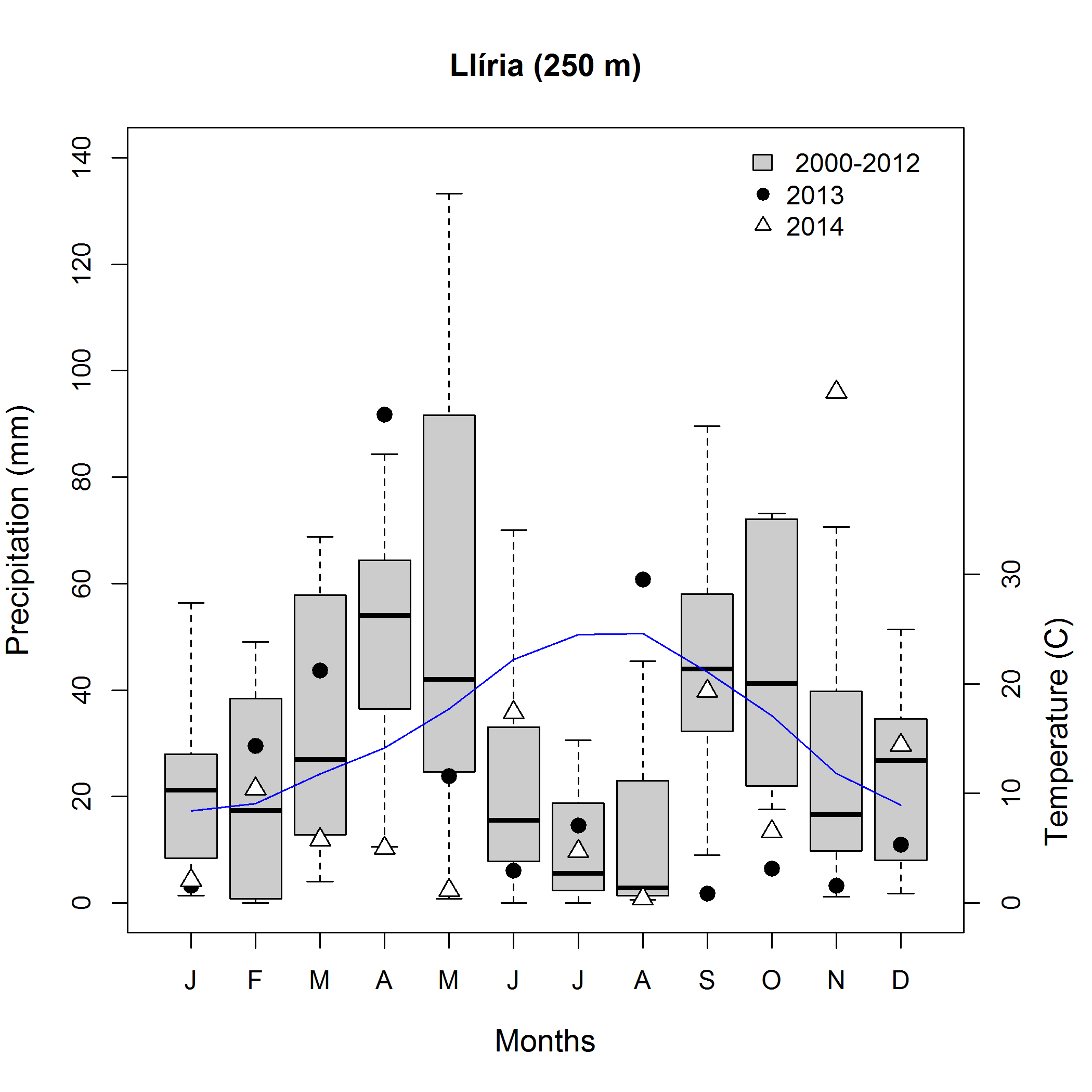

Supplement: S1 Fig — Meteorological station located at 250 m asl, between Andilla and Cortes (Valencia province). Bars show the variability in precipitation from 2000 to 2012 (left axis). Symbols are monthly precipitation for the two years sampled (2013, black circles; 2014, white triangles). Line shows mean monthly temperature (oC, right axis) for 2000 to 2012 (mean daily temperature averaged by month and year). Variability in temperature among years was much lower (not shown) than for precipitation. (TIFF) [file pone.0198951.s003.tiff]

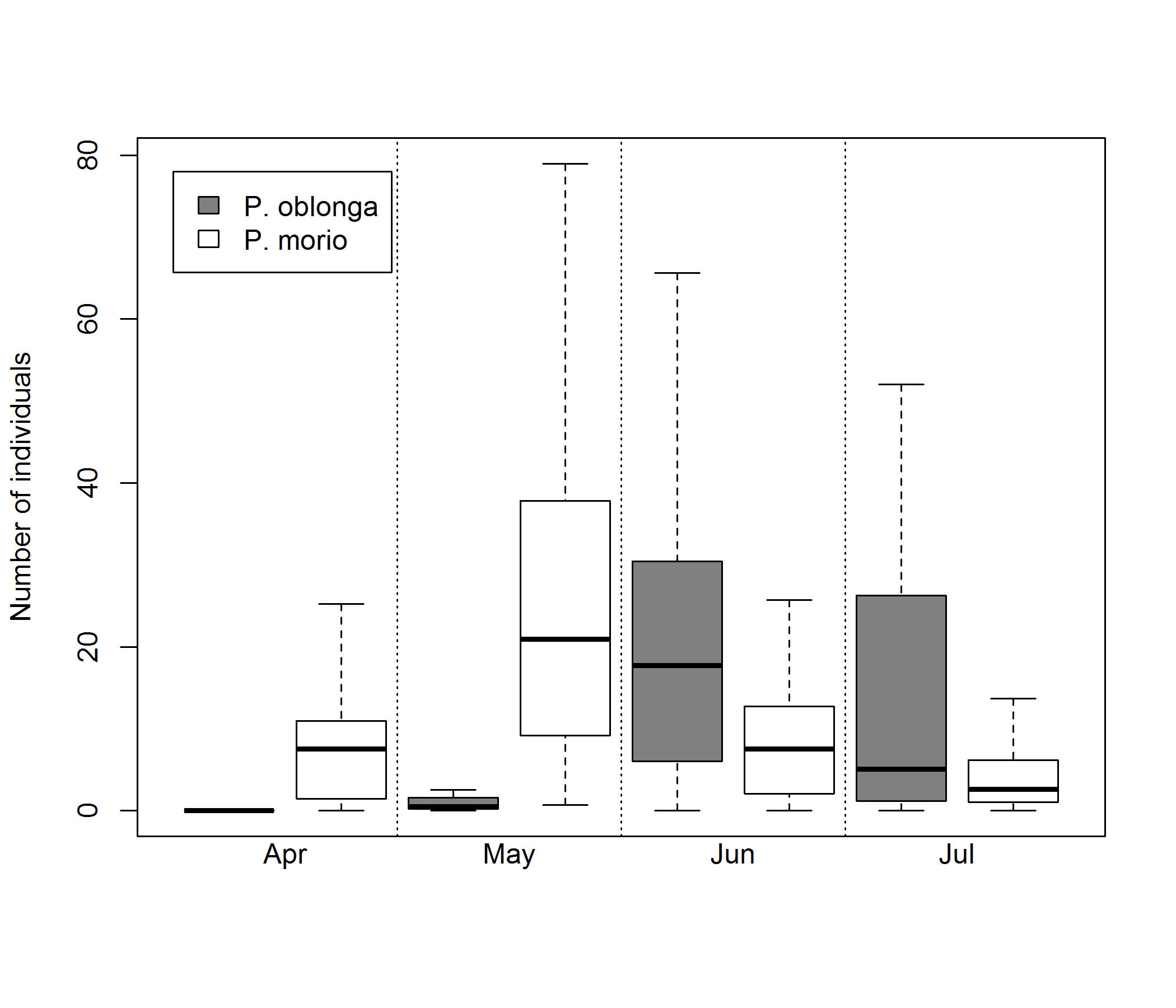

Supplement: S2 Fig — Data are mean number of individuals observed in each sampling date and for each pitfall trap (after 25 days in the field). For each species and date, variability refers to the different plots (24) and different years (2 years; except for the first date, April, that was only sampled in the second year, 2014; this April sampling was not considered in the analysis presented in the main text). This data provide an example of coexistence of closely-related species by temporal segregation of the flight activity period. P. oblonga typically occurs in drier regions than P. morio (S1 Appendix); in our study area they coexist but the P. oblonga emerged later, closer to the summer, when the weather was warmer and drier (Fig 1 main text). This temporal partitioning allows coexistence and likely contributes to the maintenance of the diversity of beetles postfire. (TIFF) [file pone.0198951.s004.tiff]

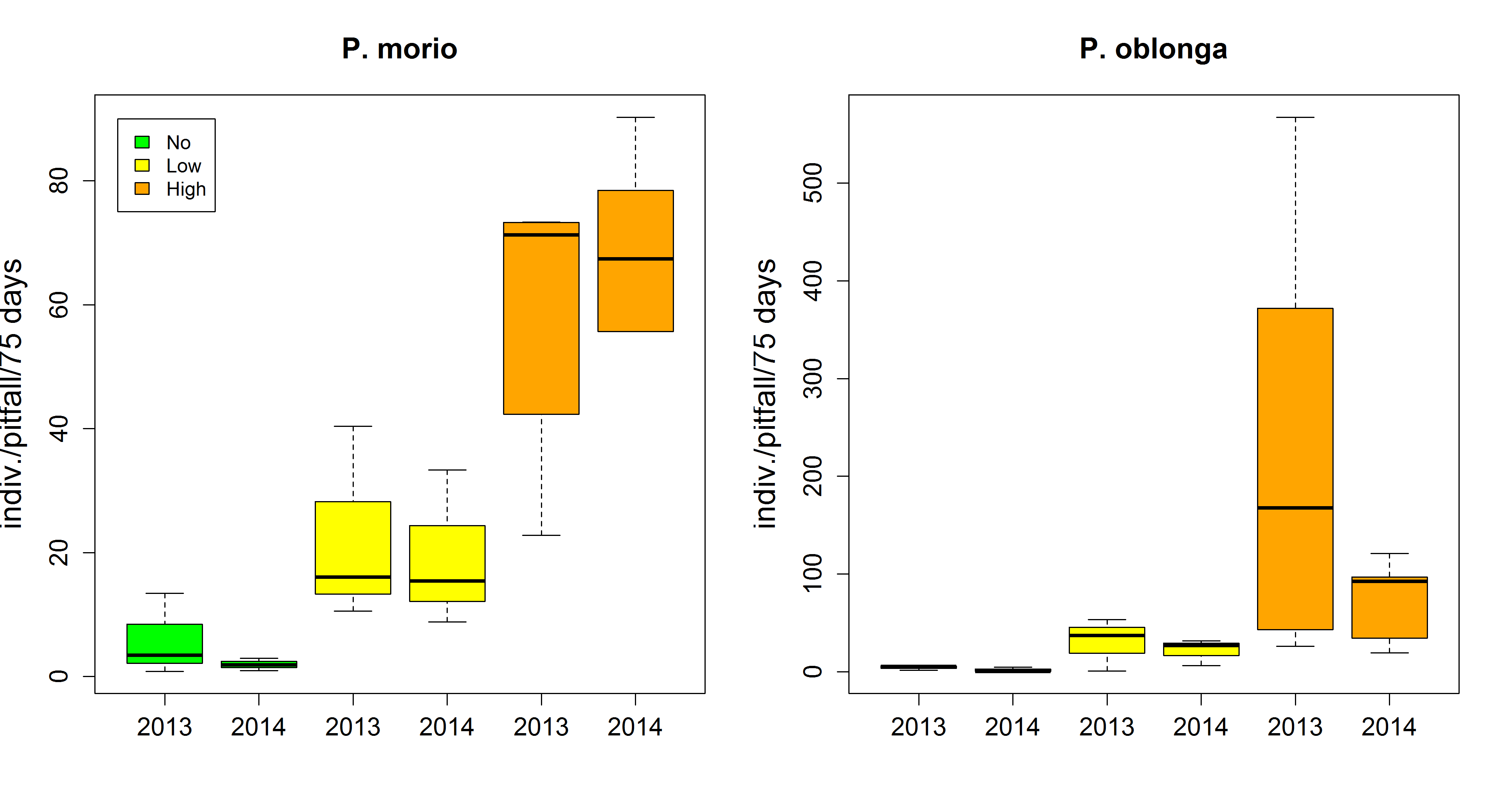

Supplement: S3 Fig — Values are mean number of individuals accumulated in a pitfall in 75 days for 2013 and 2014, in the low and high fire recurrence area (yellow and orange, respectively). Variability includes between plots and between fires; the number of plots (for each year) is 6, 12 and 6, for No, Low and High, respectively. See Table 2 for the statistical analysis. High fire recurrence correspond to plots at the center of the fire, and thus more difficult to colonise by small vertebrates (and thus more depauperated). (TIFF) [file pone.0198951.s005.tiff]

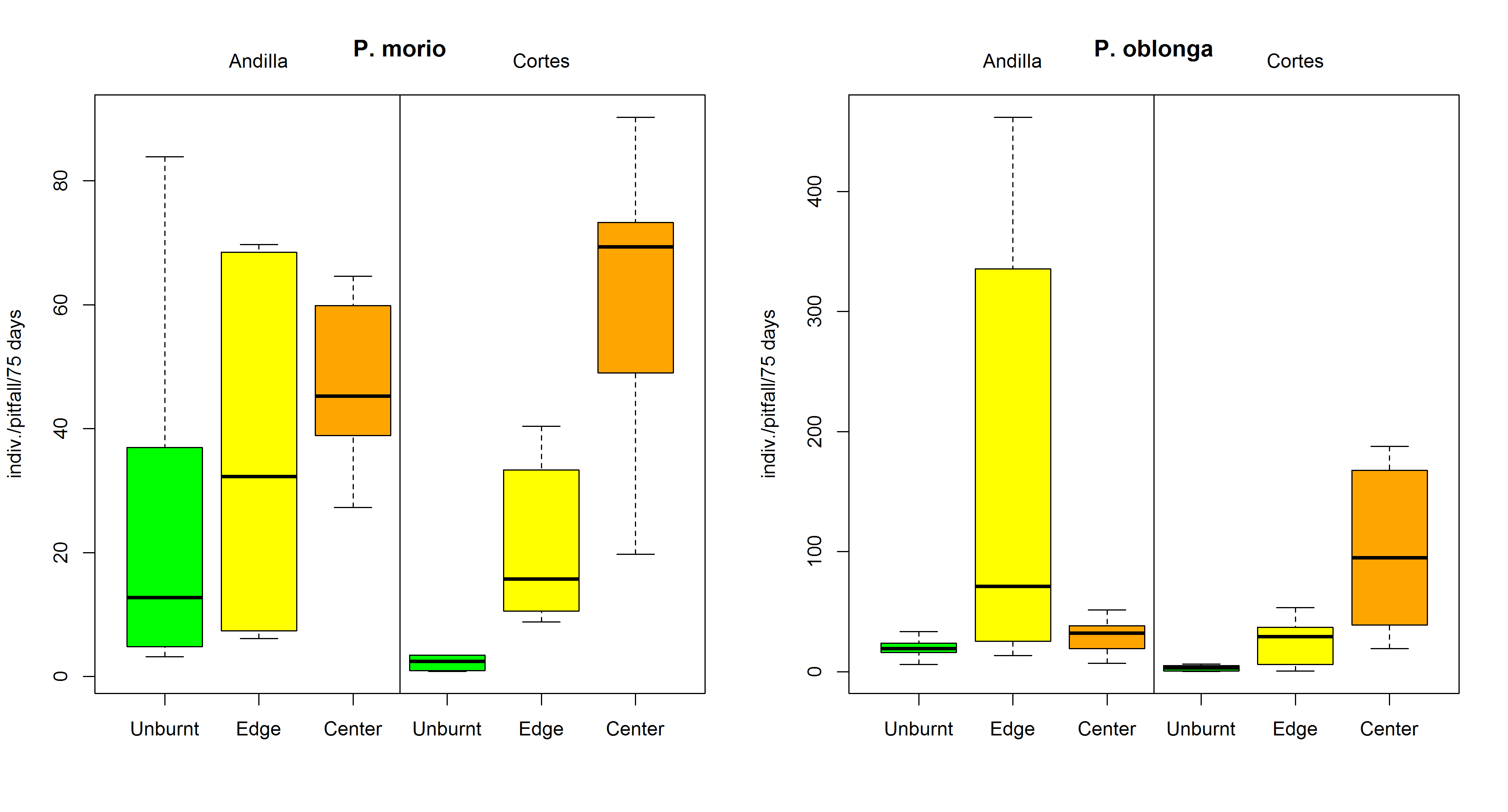

Supplement: S4 Fig — Values are mean number of individuals accumulated in a pitfall in 75 days for 2013 and 2014, in the unburnt area (green), in the burnt area close to the limit with the unburnt (Edge, yellow) and in the centre of the burnt area (Center, orange), separated by site (Andilla and Cortes); variability includes among plots and between years; the number of plots (for each year) is 6, 6 and 12, for Unburnt, Edge and Center, respectively. See Table 2 for the statistical analysis. (TIFF) [file pone.0198951.s006.tiff]
