# Supplementary material for: Fire benefits flower beetles in a Mediterranean ecosystem
Source: PLoS One. 2018 Jun 27;13(6):e0198951. doi: 10.1371/journal.pone.0198951 (PMC6021045; doi:10.1371/journal.pone.0198951)
Supplement: S1 Table — Moran’s autocorrelation values (I and p-value) for the abundance of the two Protaetia species, the two years and the two sites, and the autocorrelation of the residuals after fitting the most parsimonious model (lowest AIC, Table 2A). Significance is tested by 1000 permutations. (PDF) [file pone.0198951.s007.pdf]

**S1 Table. Spatial autocorrelation**

Moran's autocorrelation values (I and p-value) for the abundance of the two *Protaetia* species, for the two years and for the two sites, and the autocorrelation of the residuals after fitting the most parsimonious model (lowest AIC, Table 2a). Significance is tested with 1000 permutations.

| | | | Abundance | | Model residuals | |
|---|---|---|---|---|---|---|
| Site | Year | Species | I | p | I | p |
| Cortes | 2013 | PO | 0.443 | **0.033** | -0.008 | 0.321 |
| Cortes | 2013 | PM | 0.464 | **0.018** | 0.130 | 0.178 |
| Andilla | 2013 | PO | -0.387 | **0.017** | -0.161 | 0.365 |
| Andilla | 2013 | PM | 0.138 | 0.096 | -0.038 | 0.386 |
| Cortes | 2014 | PO | 0.625 | **0.003** | -0.008 | 0.308 |
| Cortes | 2014 | PM | 0.445 | **0.030** | 0.130 | 0.153 |
| Andilla | 2014 | PO | -0.145 | 0.380 | 0.132 | 0.100 |
| Andilla | 2014 | PM | 0.019 | 0.250 | -0.153 | 0.358 |
